# Interlobar pulmonary sequestration with celiac aberrant artery in an elderly patient treated with combined endovascular and video-assisted thoracoscopic approach

**DOI:** 10.1016/j.radcr.2024.05.018

**Published:** 2024-05-28

**Authors:** Alessandro Monfregola, Leda De Angelis, Rosita Comune, Francesco Arienzo, Giovanni Barbato, Mario Di Stasio, Domenico Pourmolkara, Nicola Rosano, Stefano Giusto Picchi, Michele Galluzzo, Vincenza Granata, Stefania Tamburrini

**Affiliations:** a Division of Radiology, Università degli Studi di Napoli Federico II, Naples, Italy; b Division of Radiology, Università degli Studi della Campania Luigi Vanvitelli, Naples, Italy; c Department of Interventional Radiology, Ospedale del Mare, ASL NA1 Centro, Naples, Italy; d Department of Thoracic Surgery, Ospedale del Mare, ASL NA1 Centro, Naples, Italy; e Department of Radiology, Ospedale del Mare, ASL NA1 Centro, Naples, Italy; f Department of Emergency Radiology, San Camillo Forlanini Hospital, Rome, Italy; g Division of Radiology, Istituto Nazionale Tumori IRCCS Fondazione Pascale-IRCCS di Napoli, Naples, Italy

**Keywords:** Interlobar pulmonary sequestration, Computed tomography, Embolization, Thoracic surgery, VATS, Hemoptysis, Hybrid treatment, Celiac trunk

## Abstract

Pulmonary sequestration is a rare congenital pulmonary anomaly where a portion of the lung parenchyma is supplied by an anomalous systemic artery, usually originating from the thoracic or abdominal aorta. Traditionally surgical resection and ligation of the aberrant feeding vessel are the gold standard treatments of this disease. Hybrid operations consisting in endovascular arterial embolization and surgical resection is a promising treatment option. We report a case of a 69-years-old man with symptomatic intralobular sequestration successfully treated by hybrid approach.

## Introduction

Pulmonary sequestration (PS) is a rare congenital abnormality of the lower respiratory tract, where a portion of the lung parenchyma is not in continuity with the tracheobronchial tree and it is supplied by an anomalous systemic artery [1,^2^].

PS is a relatively rare condition accounting for 0.15% to 6.4% of all congenital pulmonary malformations [3], although it is the second most common congenital lung malformation with an estimated incidence of 0.15%–1.8%. PS is divided into 2 types in intra- or extra-lobar based on the nature of their pleural covering [2]. Intralobar sequestration (ILS) accounts for 75% of cases and it is characterized by sharing the same visceral pleural lining of the native lung and by a venous drainage into the pulmonary veins. Extralobar sequestration (ELS) accounts for the remaining 25% of cases and it is represented by a mass of pulmonary parenchyma with its own visceral pleural investment outside the normal lung. ELS characteristically drains into the systemic veins, commonly the azygos or the hemi-azygos venous system and forms an accessory lobe called a “Rokitansky lobe” [2,^4^,^5^]. A clear differentiation between ILS and ELS could be often difficult in presence of a mixed type of venous drainage to both pulmonary and systemic circulations [3,^6^,^7^].

ELS is usually discovered in infancy associated with other congenital anomalies including diaphragmatic hernias and defects, congenital heart diseases and communications with the foregut [8,^9^]. Instead, ILS typically presents late in childhood or early adulthood, often discovered incidentally or because of complaints of recurrent infections and pneumonia [8,^9^]. Despite the absence of a tracheobronchial connection, bacteria may access the sequestrated lung through the pores of Kohn, colonizing the sequestration that resulting in persistent infection because of lack of bronchial drainage [10]. Rarely ILS complicated with malignancy including fibrous mesothelioma and lung carcinoma [11,^12^]. ELS is considered a childhood disease and most of the cases are diagnosed early in life. Instead, ILS incidence can be underestimated because it can discover incidentally and because it can present with recurrent pulmonary infections [2,^6^], hemoptysis, congestive heart failure, systemic air embolism [[13], [14], [15], [16] and even malignancy.

Microscopically, the lung parenchyma is replaced by a cystic appearance with chronic inflammation and fibrosis. Remnants of bronchi and bronchioles are surrounded by dense fibrous connective tissue with lymphocytic infiltration [6]. These cysts are typically lined by cuboidal or columnar epithelium; additionally, alveoli involved in the sequestration may display characteristics of hyperinflation and emphysema-like changes, along with bronchiectasis [4]. ILS presentation in adults is extremely rare [17], herein we report a case of a 69-year-old male with hemoptysis with a final CT diagnosis of bronchopulmonary sequestration who underwent combined treatment with embolization and surgery.

## Case presentation

We report a case of a 69-year-old man who presented to our emergency department complaining of cough and hemoptysis for the last 24 hours.

The patient denied chest pain, shortness of breath, and weight loss. His medical history was significant only for benign prostatic hyperplasia (BPH) in treatment with alpha-lytic drugs. Surgical history was significant for orthopedic surgeries of the left knee (meniscectomy, anterior cruciate ligament reconstruction), and left-hand tendons reconstruction after ulnar nerve damage.

He denied smoking and illicit drug use. Physical examination was unremarkable, and he was hemodynamically stable.

Laboratory results including complete blood count, complete metabolic panel, and coagulation studies showed only slightly low RBC (4.29 × 10^6/mm^3, [4.5-5.8 normal values]) with normal HCT (40.6 %) [40.0-52.0 normal values], and high CPR (1.22 mg/dL [0.0-0.5 normal values]).

**Fig. 1:**
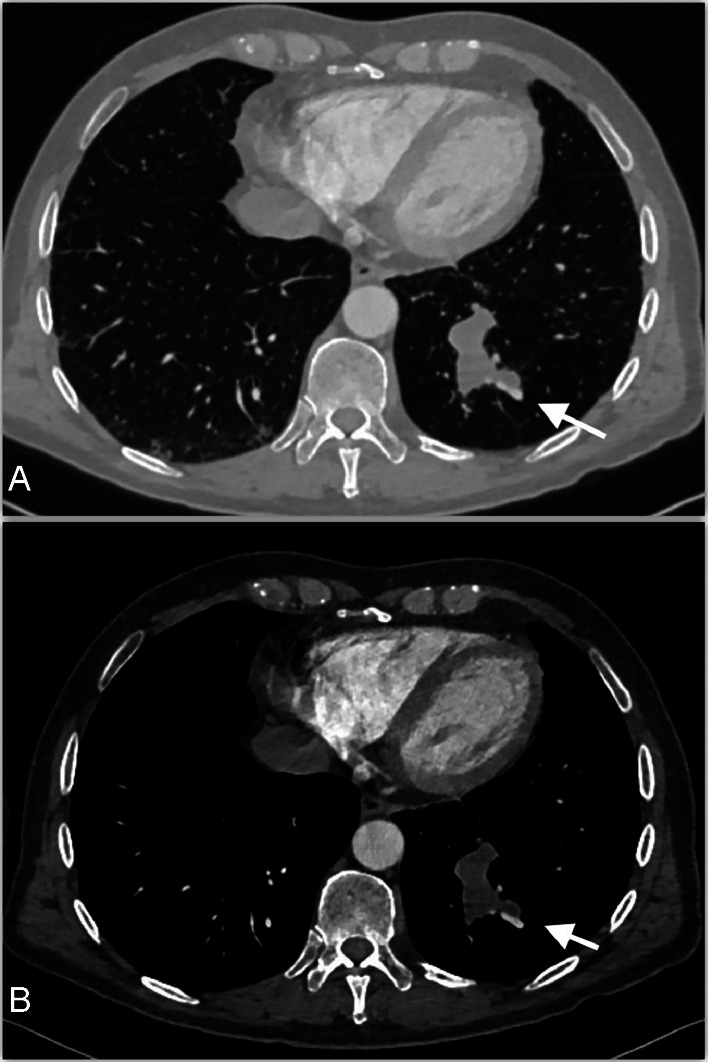
(A,B): CT with contrast axial lung window (A) and mediastinum window (B). (A) Multinodular lesions were appreciable in the left lower lobe with hyperinflation (air trapping) of adjacent lobules (arrow). (B) CT showed multinodular confluent markedly hypodense lesions with no air fluid levels in the left lower lobe (arrow).

The patient underwent thorax Angio CT with 90 mL intravenous contrast injected at 3.5 mL/s (Iopamiro 370 mg/mL, Bracco Inc.). CT was acquired in non-contrast, arterial, portal, and delayed phase. Multiple nodular confluent hypodense lesions with multicystic appearance and without air-fluid levels were detected in the left lower lobe. No evident communication between the cystic lesions and the segmental bronchus was appreciable. Diffuse perilesional hyperlucencies suggesting air trapping were detected (Fig. 1) in the basal segments.

**Fig. 2:**
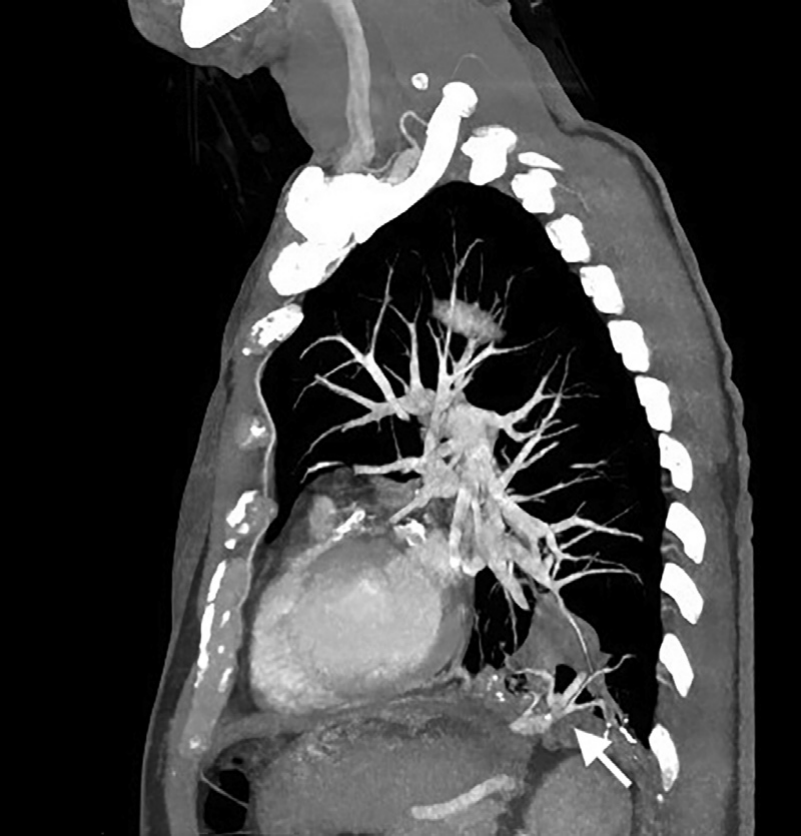
Sagittal MIP (maximum intensity projection) with intravenous contrast. Aberrant feeding artery with subdiaphragmatic origin was appreciable (white arrows).

**Fig. 3:**
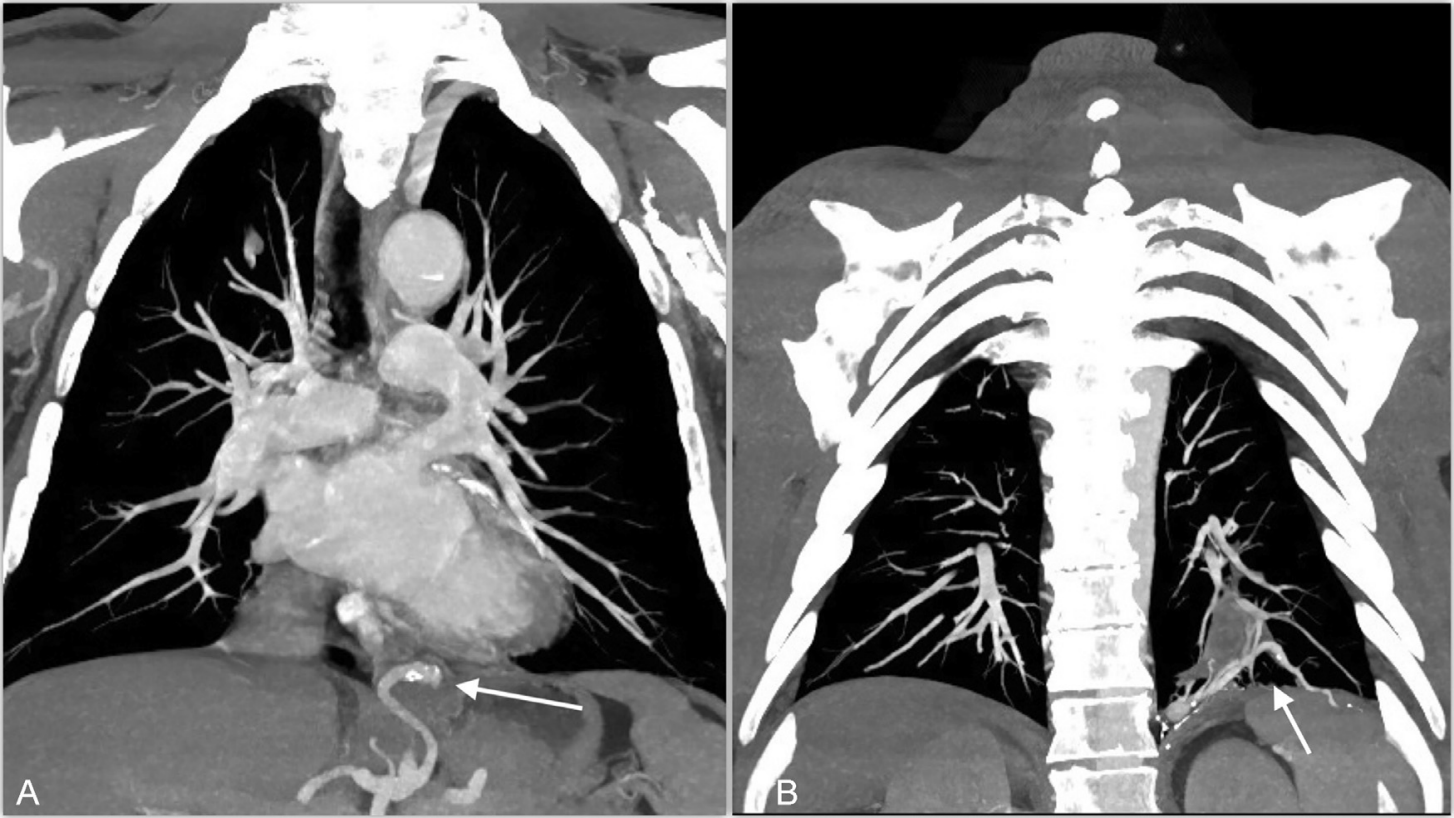
(A,B): coronal view MIP CT showed the arterial aberrant branch originating from the celiac trunk, passing through the diaphragm and suppling the left lower lobe (arrows).

The multicystic lesion was supplied by an anomalous systemic arterial vessel that raised directly from the celiac trunk. The artery was aneurysmatic (>9 mm), with elongated and tortuous course, and presented parietal calcifications and multiple thrombotic intraluminal defects (Figs. 2 and 3).

**Fig. 4:**
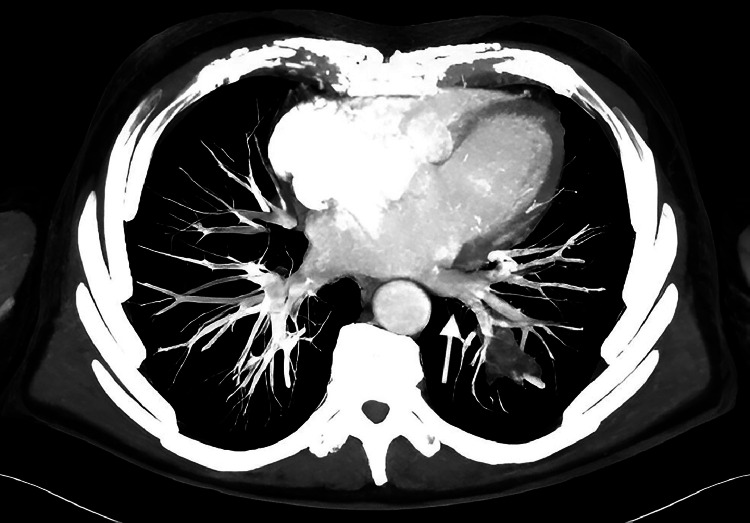
CT axial MIP demonstrated that lesion's venous drainage run from a pulmonary vein to left atrium (arrow).

The venous drainage was directed in the left atrium through a pulmonary vein (Figs. 4). A CT diagnosis of intrapulmonary sequestration with anomalous arterial supply from the celiac axis was formulated. The patient was admitted to the hospital and considering the aneurysmatic feeding artery, a combined treatment was planned.

**Fig. 5:**
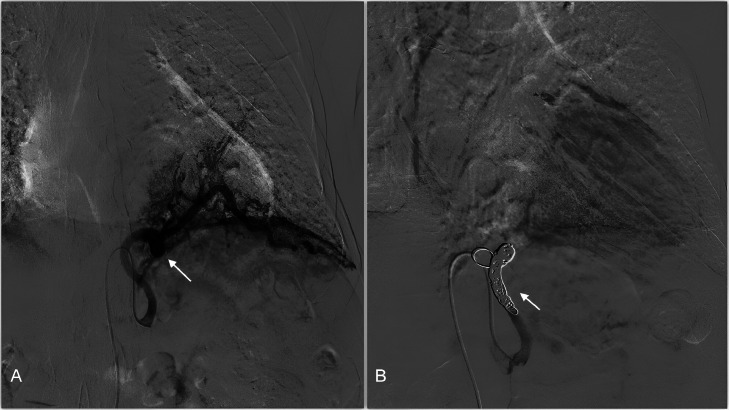
(A,B): (A) Digital subtraction angiography demonstrated aberrant left lower lobe vessel arising from celiac plexus (white arrow). (B) Aberrant left lower lobe vessel post embolization using five 6-12 mm embolization coils with satisfactory occlusion of the aberrant artery (white arrow).

First the patient underwent angiography. At angiography, the right common femoral artery was accessed for digital subtraction angiography, which confirmed a single aneurysmatic and tortuous feeding artery originating from the celiac axis coursing above the diaphragm toward the left lower lobe with numerous abnormal corkscrew-type arteries. The feeding artery was supra-selectively catheterized and embolized with five 6-12 mm embolization coils. Post embolization angiogram demonstrated successful occlusion of the feeding artery. A 6-French Angio-Seal (St. Jude Medical Inc.) closure device was deployed in the right common femoral artery (Fig. 5).

Sequently, the patient underwent left video-assisted thoracoscopic surgery (VATS) with two access ports. On introduction of optics, there was absence of adhesions and effusion. The feeder artery was thrombosed and recognizable by the presence of coils. Section of the pulmonary ligament, preparation section and suture of the celiac artery was performed. Isolation, section and suture of the pulmonary vein, of the arterial branches and bronchus for the lower lobe by 30-45-60 mm Endo Gia System (Medtronic Inc.).

Station 8 and 9 lymphadenectomies, adjacent to the celiac artery were performed. Accurate hemostasis was made.

**Fig. 6:**
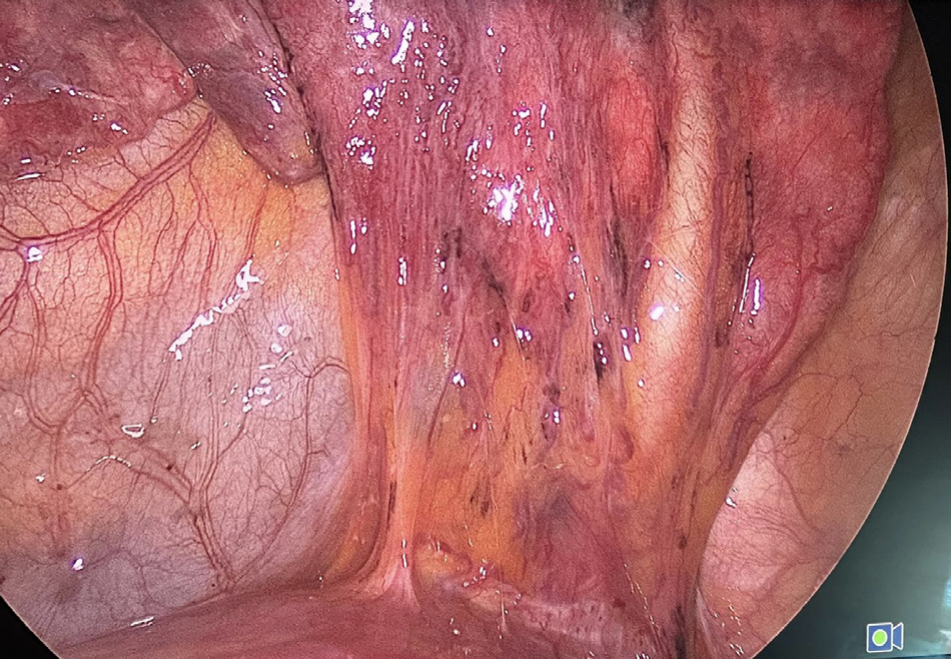
Intraoperative view of wedge resection of the lower lobe of left lung.

Placement of a chest drain, control of re-expansion of residual lung parenchyma, plane closure of surgical incisions was performed. The resected sequestrum measured 19 × 13 × 5 cm (Fig. 6). At histology, the lung sequestrum showed multiple fluid filled bronchiectasis surrounded by dense fibro-necrotic connective tissue with lymphocytic infiltration. Surrounding parenchyma exhibited atelectasis, congestion, and erythrocyte extravasation, with hyperplastic areas, cystic dilatation, and focal bronchial obliteration. Pathology report identifies a diagnosis of pulmonary bronchiectasis complicated by bronchiolitis obliterans—organizing pneumonia as a late outcome of chronic interstitial pneumonia. Areas of reactive hyperplasia with anthracosis were detected within the resected lymph nodes. The postoperative course was uneventful, and the patient was discharged on the sixth postoperative day.

## Discussion

Developmental lung anomalies encountered in adulthood can be divided into 3 broad categories: bronchopulmonary (lung bud), vascular anomalies and combined lung, and vascular anomalies [8,^9^,^18^]. PS is characterized by no direct connection to the normal bronchial tree and an arterial systemic supply. Intralobar sequestration is more common than extralobar sequestration and can be detected at any age, frequently found incidentally on an abnormal chest radiograph [2]. It can be asymptomatic and it is usually heralded by the presence of recurrent or chronic pneumonia, although some patients may present in heart failure, or with hemoptysis [2]. ILS has no predilection for gender and almost always involves the lower pulmonary lobes in as many as 98% of cases and is more typically located in the medial or posterior basal segments of the left lower lobe. In general, the left side of the chest is affected in about 60% of cases [2,^6^,^19^]. Arterial supply of ILS has been reported from the descending thoracic aorta or abdominal aorta, although many other arterial sources to the sequestered lobe have been documented (subclavian arteries, internal thoracic arteries, the arteries feeding the chest wall, and coronary arteries). The venous drainage is via the pulmonary veins [2]. Elevated CA10-9 has been reported in pulmonary sequestration, although the pathomechanism is not clear. Different hypothesis has been made: CA 19-9 might be synthesized and secreted by normal bronchial epithelial cells in the sequestrated lung or lung epithelial cells chronically infected with Aspergillus or other pathogens cause the proliferation of bronchial epithelial cells and synthesis of CA19-9 [20]. Bronchoscopy has no role in diagnosis of PS. Thorax X-ray may show an homogenous opacity (appearing as a solitary nodule or uniformly dense mass with smooth or lobulated contours) or a patchy consolidation with irregular margins that is typically located in the posterior-basal aspect of the left lower lobe [10,^12^,^18^,^21^] that requires second level imaging with Angio CT.

The definitive diagnosis of PS is histological but preoperative diagnosis can be achieved by the identification of a systemic arterial supply of the pulmonary lesions [14,^22^]. In this scenarios, CT and MR can assess a definitive diagnosis by noninvasive identification of systemic arterial supply [24] and multi-detector computed tomography (MDCT) of the chest is the diagnostic imaging modality of choice for all DLAs [23,^24^] with the exception of pulmonary venous anomalies, which are better evaluated with magnetic resonance imaging (MRI).

The CT features of PS are variable, it can present as a mass, cystic lesions, cavitary lesions, pneumonic lesions, and bronchiectasis and for these reasons the diagnosis can be challenging with a high rates of misdiagnosis [8,^14^,^25^]. Mass form of PS can present homogenous or heterogenous enhancement. Instead in cystic form of PS the cysts can be air filled, fluid filled or containing air-fluid level. The air fluid level could be explained by a fistulae bronchial communication determined by recurrent infections [26]. In our case a multicystic form of intralobular ILS was detected with no air fluid levels and no communication with the bronchial tree. Differential diagnosis should include lung abscesses, necrotizing pneumonia and mycetomas [21,^25^]. The variable imaging pattern of PS requires particular attention in identifying the anomalous systemic arterial supply that can aid to a definitive diagnosis and CT Angiography represents the making of choice in order to identify an aberrant systemic artery and parenchymal alterations [14,^27^]. Systemic artery supplying the PS usually arises from the lower thoracic aorta, however, it can also arise from the upper abdominal aorta, as in our case, and rarely from other systemic arteries [22,^26^,^28^,^29^]. The diameter of systemic arteries is variable [14,^22^,^30^]. CT multiplanar reformations and volume rendering (VR) reconstructions can be helpful to increase the diagnostic confidence and overall accuracy, aiding treatment planning, and improving communication with both clinicians and patients [24,^27^,^31^]. Modern dual energy CT scanners can deliver a radiation dose that is equivalent or less compared with conventional single-energy CT equipment and allow optimizing tissue and vessel contrast, avoiding true precontrast scans, assessing functional parameters such as pulmonary perfusion, and improving the detection of perfusion within vascular malformations [32].

In our case the aberrant systemic artery appeared extremely tortuous and dilated (9 mm) and MPR and VR reconstruction allowed detailed anatomical information of the origin and the course originating from the celiac trunk.

Other CT findings in PS that can support the diagnosis are emphysematous changes or hyperlucency at the margin of the lesion are characteristically determined by collateral air drift and air trapping due to the absence of a normal bronchial connection [14,^33^].

PS is commonly resected and with the accelerated development of minimally invasive techniques, video-assisted thoracoscopic surgery (VATS) has been widely used in the treatment [34,^35^].

Surgical resection is recommended because of the likelihood of recurrent infection, the need for larger resection if the sequestration becomes chronically infected, the risk of hemorrhage, and the reported development of malignancy, even in asymptomatic patients [[36], [37], [38]. The treatment for symptomatic patients with PS has always been surgical resection [39].

Lobectomy is usually performed, although wedge resection can be preferred for smaller lesions that are distant from the pulmonary hilum and with a significant portion of surrounding normal lung tissue [40]. Transcatheter embolization is considered a minimally invasive technique that has been adopted for definitive treatment of PS carrying a lower morbidity than surgical resection [41]. Endovascular occlusion of the arterial supply can reduce blood flow to the sequestered tissue, leading to necrosis, fibrosis, and progressive involution, and hence can represent a useful presurgical approach to minimize the risk of intraoperative bleeding, as well as an alternative to surgery in selected cases, including small PS (<3 cm) and complications such as acute hemoptysis [[41], [42], [43], [44]. Moreover, the sequestration's vascular supply is often friable due to inflammation from repeated infection, and embolization has been demonstrated to reduce the risk of intraoperative hemorrhage [28,^41^,^45^,^46^].

In our case hybrid operation consisting of embolization followed by surgical resection reduced the risk of intraoperative hemorrhage with potentially higher safety and effectiveness than single resection or embolization [40,^41^,^43^,^44^,[47], [48], [49].

## Conclusions

ILS in elderly patients (aged >60 years) is rarely reported. Knowledge of ILS CT appearance and aberrant arterial supply may support radiologists to reach a preoperative definitive diagnosis. Hybrid treatment with transarterial embolization and VATS appears a safe approach reducing intra-operative bleeding risk.

## Patient consent

Written informed consent was obtained from the patient for publication of this case report and accompanying images. A copy of the written consent is available for review by the Editor-in-Chief of this journal on request.
